# Roles of Interleukin‐31 in Allergen‐Driven Cough: Linking Protease‐Activated Receptor‐2 Activation and Vagal Ganglion Sensitization in a *Dermatophagoides farinae*‐Induced Asthma Model

**DOI:** 10.1002/brb3.71731

**Published:** 2026-09-04

**Authors:** Takayoshi Miyamoto, Chiharu Ohira, Mao Kaneki, Mariko Komuro, Mana Ichikawa, Ibuki Yasuda, Yoshiichi Takagi, Tomoki Fukuyama

**Affiliations:** ^1^ School of Veterinary Medicine Azabu University Sagamihara‐shi Kanagawa Japan; ^2^ Japan SLC, Inc. Hamamatsu City Shizuoka Japan; ^3^ Center for Human and Animal Symbiosis Science Azabu University Sagamihara‐shi Kanagawa Japan

**Keywords:** cough, IL‐31, PAR‐2, vagal ganglia

## Abstract

**Background:**

Chronic cough, a distressing symptom of allergic asthma, possibly arises from the abnormal activation of airway‐projecting sensory neurons. Levels of interleukin (IL)‐31, a Th2 cytokine known for its neuronal effects in pruritus, are elevated in allergic disorders; however, its role in asthma‐related cough remains unclear. Therefore, in this study, we aimed to determine the mechanism by which IL‐31 influences cough‐associated neuronal sensitization using a *Dermatophagoides farinae* (*Derf*)‐induced asthma model.

**Methods:**

Wild‐type (WT) and *Il31* knockout (KO) C57BL/6N mice were intranasally administered the *Derf* extract repeatedly. Lung tissues, bronchoalveolar lavage fluid, and hilar lymph nodes were analyzed via histology, flow cytometry, enzyme‐linked immunosorbent assay, and quantitative polymerase chain reaction. Vagal and dorsal root ganglia were assessed via immunofluorescence assay and Ca^2^^+^ imaging.

**Results:**

Protease‐activated receptor (PAR)‐2 activity was evaluated using the agonist SLIGRL‐NH_2_. *Derf* upregulated *Il31* levels in airway tissues. Notably, immune and histological parameters were comparable between WT and *Il31* KO mice, indicating that IL‐31 deficiency did not alter Th2‐driven inflammation. In contrast, *Derf*‐challenged WT mice exhibited increased *Il31ra* and *F2rl1* (PAR‐2) levels and PAR‐2‐positive neuron proportions in vagal ganglia, changes that were not observed in *Il31* KO mice. Ca^2^^+^ imaging confirmed that PAR‐2 responsiveness to SLIGRL‐NH_2_ was enhanced only in WT asthma model mice.

**Conclusion:**

Overall, IL‐31 modulated the neuronal, rather than immune, components of allergic asthma by enhancing the PAR‐2‐dependent excitability of vagal sensory neurons. These findings highlight the IL‐31–PAR‐2–vagal pathway as the potential mechanism of and therapeutic target for allergen‐driven cough.

## Graphical abstract

In a *Dermatophagoides farinae*‐induced asthma model, IL‐31 deficiency did not substantially alter airway inflammation or immune responses but prevented the allergen‐induced enhancement of PAR‐2 expression and responsiveness in vagal sensory neurons. These findings identify IL‐31 as a selective modulator of neuronal sensitization in allergic asthma.

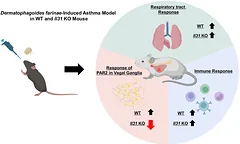

**Abbreviations**
BALF: bronchoalveolar lavage fluid
*Derf*
: *Dermatophagoides farinae*
DRGs: dorsal root ganglia
ELISA: enzyme‐linked immunosorbent assay
HDM: house dust mite
IL‐31: interleukin‐31
KO: knockout
PAR‐2: protease‐activated receptor‐2
TSLP: thymic stromal lymphopoietin
VGs: vagal ganglia
WT: wild‐type

## 1 Introduction

Allergic asthma is a heterogeneous inflammatory airway disease characterized by airway hyperresponsiveness, mucus overproduction, and episodic symptoms such as wheezing, dyspnea, and cough. Specifically, chronic cough often persists despite standard anti‐inflammatory therapy and remains a major determinant of disease burden, interfering with sleep, work, and social interactions, thereby substantially impairing the quality of life (Morice et al. 2020; Naqvi et al. 2023). Although airway inflammation and bronchoconstriction are well‐established features of asthma, accumulating evidence suggests that cough is frequently sustained by the aberrant activation and sensitization of airway sensory pathways, rather than by inflammation alone (Mazzone et al. 2018).

The cough reflex is primarily mediated by vagal afferent neurons, whose cell bodies reside in the jugular and nodose vagal ganglia (VGs), with additional contributions from the dorsal root ganglia (DRGs) that convey chest wall sensations and the urge to cough (Drake et al. 2021). These neurons express various sensory receptors, including transient receptor potential (TRP) channels (e.g., TRPV1 and TRPA1) and protease‐activated receptor (PAR)‐2, a G‐protein‐coupled receptor activated by serine proteases (Amadesi et al. 2004; Dai et al. 2007). Allergen proteases, such as those from *Dermatophagoides farinae* (*Derf*), activate PAR‐2, inducing epithelial cytokine release and neuronal hyperexcitability. This process possibly lowers the activation threshold of cough pathways, contributing to chronic cough hypersensitivity in asthma (Asokananthan et al. 2002; Kupari et al. 2019).

Interleukin (IL)‐31, a Th2‐derived cytokine, signals through a heterodimeric receptor composed of the IL‐31 receptor A (IL‐31RA) and oncostatin M receptor β (Cornelissen et al. 2012; Dillon et al. 2004). It a critical mediator of neuroimmune interactions, particularly in atopic dermatitis, where it drives pruritus via direct activation of IL‐31RA‐expressing sensory neurons (Cevikbas et al. 2014; Ruzicka et al. 2017). IL‐31 signaling engages the mitogen‐activated protein kinase and signal transducer and activator of transcription pathways to induce cytokines, chemokines, and neuronal sensitization. The success of IL‐31RA‐targeted therapies in pruritic disorders further underscores its dual immunomodulatory and neuromodulatory roles (Kabata and Artis 2019).

IL‐31 expression and serum levels are elevated and correlated with disease severity in asthma (Huang et al. 2023; Lai et al. 2016). Experimental data suggest that IL‐31RA signaling promotes airway smooth muscle excitability and airway hyperresponsiveness (Akkenepally et al. 2023). However, the mechanistic role of IL‐31 in the pathophysiology of asthma remains ambiguous. Additionally, whether IL‐31 acts predominantly via immune pathways, epithelial interactions, or neuronal circuits underlying key symptoms such as cough remains unclear.

Considering the established role of IL‐31 in sensory modulation and involvement of PAR‐2 in cough hypersensitivity, we hypothesized that IL‐31 contributes to cough generation in allergic asthma by enhancing the PAR‐2‐mediated excitability of airway sensory neurons. To verify this, we established *Derf*‐induced asthma models in wild‐type (WT) and *Il31*‐deficient mice, combining classical immunological analyses with direct neuronal assessments of VG responsiveness. Using this approach, we aimed to delineate the relative contributions of immune and neuronal IL‐31 signaling and identify the potential neuroimmune mechanism linking Type 2 inflammation to allergen‐driven cough.

## 2 Materials and Methods

### 2.1 Patient Data Analysis

In silico analysis of the GSE73482 dataset (Affymetrix Human Gene 1.0 ST) from the National Center for Biotechnology Information Gene Expression Omnibus database was performed, followed by data processing in R (oligo) using RMA, with expression values reported as log2 RMA. Sample grouping followed the Gene Expression Omnibus SOFT metadata and filename suffixes (“_C.CEL” = medium; “_H.CEL” = house dust mite [HDM]). For HDM‐stimulated comparisons, we analyzed 24 non‐asthmatic (nt_HDM) and 22 asthmatic (asthma_HDM) samples. Probeset annotation was performed using the hugene10sttranscriptcluster.db package.

### 2.2 Animals and Ethics

Six‐week‐old female C57BL/6N WT and *Il31*‐deficient (*Il31* knockout [KO]) mice were obtained from Japan SLC, Inc. (Shizuoka, Japan). *Il31* KO mice were generated as previously described (Takamori et al. 2018). All mice were housed under standard conditions (12/12‐h light/dark cycle, 22°C ± 3°C, and 55% ± 15% humidity), with ad libitum access to certified pellet diet and water. A maximum of four mice were housed per cage. All procedures adhered to the Azabu University (Kanagawa, Japan) Animal Care and Use Program (approval no. 230518‐6) and National Guidelines for the Care and Use of Laboratory Animals.

### 2.3 *Derf*‐Induced Mouse Model of Allergic Asthma

The mice were sensitized and intranasally challenged with the *Derf* extract (1 mg/mL; ITEA Inc., Tokyo, Japan) under isoflurane (FUJIFILM Wako Pure Chemical Corporation, Osaka, Japan) anesthesia. Sensitization was performed on Days 0, 7, and 14 with 25 µg *Derf* in 25 µL phosphate‐buffered saline (PBS). Five days after the last sensitization, the mice were challenged with 25 µg *Derf* in 25 µL PBS for 3 consecutive days (Days 18–20). Twenty‐four hours after the final challenge, the mice were euthanized under isoflurane anesthesia, and samples, including serum, bronchoalveolar lavage fluid (BALF), hilar lymph node (LN), lung, VG, and DRG samples, were collected for analysis.

### 2.4 Quantitative Polymerase Chain Reaction

Tissues were homogenized using a bead‐beater homogenizer (µT‐12; TAITEC CORPORATION, Tokyo, Japan), and total RNA was extracted using the NucleoSpin RNA kit (TaKaRa Bio Inc., Shiga, Japan). Reverse transcription of 500 ng RNA was performed using the PrimeScript RT Master Mix (TaKaRa Bio Inc.). Gene expression was quantified using the PowerUp SYBR Green Master Mix (Thermo Fisher Scientific, Kanagawa, Japan) on the CFX Duet Real‐Time PCR System (Bio‐Rad Laboratories Inc., Tokyo, Japan). Real‐time PCR was performed under the following thermal cycling conditions: initial denaturation at 95.0°C for 3 min, followed by 40 cycles of denaturation at 95.0°C for 10 s and annealing/extension at 60.0°C for 30 s, with fluorescence data collected at the end of each annealing/extension step. After amplification, melt curve analysis was performed by holding the reaction at 65.0°C for 5 s and then increasing the temperature stepwise to 95.0°C in 0.5°C increments, with continuous fluorescence acquisition, to confirm amplicon specificity. Target gene expression was normalized to β‐actin, and relative expression was calculated using the 2^−ΔΔCt^ method. All primer sequences are listed in Table S1.

### 2.5 Histology and Immunofluorescence Assay

The lungs were fixed in 10% formalin, embedded in paraffin, sectioned at 5 µm, and stained with hematoxylin and eosin (FUJIFILM Wako Pure Chemical Corporation). VGs were enzymatically dissociated in 1.25 mg/mL dispase II and 1.25 mg/mL collagenase type I (FUJIFILM Wako Pure Chemical Corporation). Peripheral neurons were seeded on a glass‐bottom dish coated with laminin and poly‐*L*‐lysine (FUJIFILM Wako Pure Chemical Corporation) and incubated for 24 h. After fixing with 10% formalin, the cells were blocked with Blocking One Histo (NACALAI TESQUE, INC. Kyoto, Japan) and incubated overnight with the anti‐PAR2 primary antibody (Santa Cruz Biotechnology, Inc., Dallas, Texas, USA) in PBS (FUJIFILM Wako Pure Chemical Corporation) containing Tween 20 and a 5% blocking solution at 4°C. Alexa Fluor 568‐conjugated secondary antibody (Thermo Fisher Scientific) was used for detection, and the nuclei were counterstained with 4',6‐diamidino‐2‐phenylindole (BD Biosciences, Tokyo, Japan). Finally, images were acquired using the ECLIPSE TE300 microscope (Nikon Solutions Corporation, Tokyo, Japan), and PAR2‐positive cells were quantified in 10 random fields per sample.

### 2.6 Flow Cytometry

Single‐cell suspensions were prepared from hilar LNs as previously described (Aihara et al. 2020; Ookawara et al. 2020), and total cell numbers were determined using the CellDrop Cell Counting System (DeNovix Inc., Wilmington, Delaware, USA). To prevent nonspecific antibody binding, 1 × 10^6^ cells were preincubated with 1 µg of mouse Fc Block (Miltenyi Biotec K.K., Tokyo, Japan) before surface staining. The cells were subsequently incubated with the following monoclonal antibodies: PE/cyanine7‐conjugated anti‐mouse CD3, PE‐conjugated anti‐mouse CD4, PE‐conjugated anti‐mouse CD11b, APC‐conjugated anti‐mouse CD11c, PerCP/cyanine5.5‐conjugated anti‐mouse CD19, APC‐conjugated anti‐mouse CD44, APC/cyanine7‐conjugated anti‐mouse CD62L, and fluorescein isothiocyanate‐conjugated anti‐mouse MHC class II (BioLegend Inc., San Diego, California, USA; Miltenyi Biotec K.K.) antibodies. Moreover, 4',6‐diamidino‐2‐phenylindole (BioLegend Inc.) was used for cell viability assessment. After washing, the cells were analyzed using the BD FACSAria III Cell Sorter (BD Biosciences).

### 2.7 Enzyme‐Linked Immunosorbent Assay (ELISA)

BALF was collected via tracheal cannulation and lavaged thrice with 1 mL PBS containing 0.1% bovine serum albumin. IL‐33 and thymic stromal lymphopoietin (TSLP) levels in BALF were measured using commercial ELISA kits (R&D Systems, Minneapolis, Minnesota, USA). Serum total IgE levels were also quantified using an ELISA kit (Thermo Fisher Scientific). Target protein concentrations were measured using sandwich ELISA. Ninety‐six‐well plates were coated with capture antibody and incubated overnight at room temperature (IL‐33 and TSLP) or at 4°C (IgE). After washing with wash buffer, samples and standards were added to the wells and incubated for 2 h (IL‐33 and TSLP) or 1 h (IgE). After an additional wash, detection antibody was added and incubated for 2 h (IL‐33 and TSLP) or 1 h (IgE). The wells were then incubated with streptavidin‐HRP. After a final wash, TMB substrate solution was added for color development, and the reaction was stopped with hydrochloric acid. Absorbance was measured at 450 nm using a Multiskan SkyHigh microplate reader (Thermo Fisher Scientific). Sample concentrations were determined by interpolation from standard curves generated for each analyte.

### 2.8 Calcium Imaging

VG neurons were loaded with Fura‐2 AM (30 min; 37°C) and imaged using a fluorescence microscope under continuous perfusion with HEPES‐buffered saline (115 mM NaCl, 5.4 mM KCl, 1.8 mM CaCl_2_, 0.8 mM MgCl_2_, 20 mM HEPES, and 13.8 mM glucose; pH 7.4; 40°C). Subsequently, the neurons were stimulated with 100 µM SLIGRL‐NH_2_ (FUJIFILM Wako Pure Chemical Corporation), and intracellular Ca^2^^+^ responses were quantified. The percentage of responsive neurons was calculated for each condition. Responsive (“positive”) cells were defined as neurons showing an increase in the fluorescence ratio (340/380 nm) after SLIGRL‐NH_2_ stimulation, and the percentage of positive cells was calculated as the number of responding neurons divided by the total number of neurons analyzed in each group. Data were pooled from seven WT mice and eight *Il31* KO mice.

### 2.9 Statistical Analyses

Data are represented as the mean ± standard deviation. Gene expression levels were compared using an unpaired *t*‐test. Calcium‐imaging response proportions were compared among groups using Fisher's exact test (two‐sided). Other data were analyzed via two‐way analysis of variance with multiple comparisons. Statistical analyses were conducted using GraphPad Prism v10 (GraphPad Software, San Diego, California, USA). Statistical significance was set at *p* < 0.05.

## 3 Results

### 3.1 HDM‐Sensitization Induces Th2 Inflammatory Responses and Il31 Upregulation in Patients With Asthma

**FIGURE 1:**
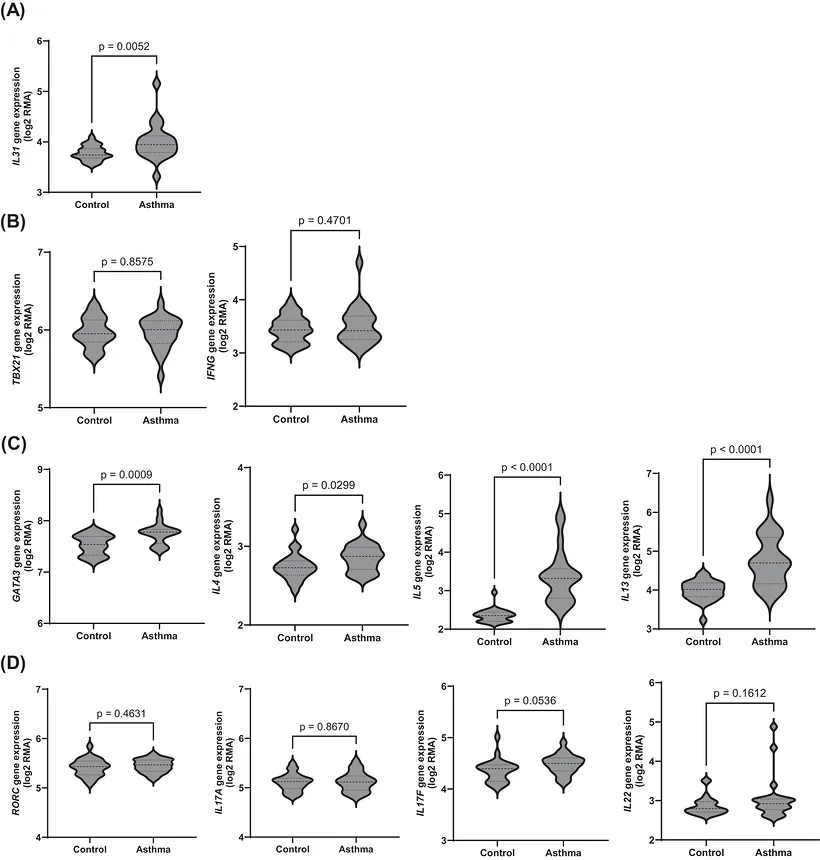
Th2‐dependent responses and elevated interleukin‐31 (*IL31*) gene levels in house dust mite (HDM)‐sensitized patients with asthma. Public microarray data from the CD4^+^ T cells of HDM‐sensitized patients with asthma were analyzed for the levels of *Il31* and Th1 (B), Th2 (A and C), and Th17 (D) representative genes. Data represent mean ± SEM (*n* = 24 [control] and 22 [asthma]). Statistical significance was assessed using an unpaired *t*‐test.

Allergic asthma is driven by environmental allergens derived from various sources, such as HDMs, cockroaches, dogs, and cats (Wright and Phipatanakul 2014). Among these allergens, HDMs are considered as the most important triggers (Chauhan et al. 2020; Hsin et al. 2024). To evaluate the effects of HDMs under asthmatic conditions, we analyzed the gene expression profiles of CD4^+^ T cells from HDM‐sensitized patients with asthma using microarray data from the National Center for Biotechnology Information Gene Expression Omnibus Microarray database. Comparison of the expression levels of representative Th1 (Figure 1B), Th2 (Figure 1A,C), and Th17 (Figure 1D) genes revealed that only Th2‐associated gene levels were upregulated in HDM‐sensitized patients (Figure 1A,C). Consistent with a Th2‐type response, IL31 levels were also increased in HDM‐sensitized patients (Figure 1A).

### 3.2 *Derf*‐Induced Allergic Asthma Model Mice Show Elevated *Il31* Levels in Airway Tissues

**FIGURE 2:**
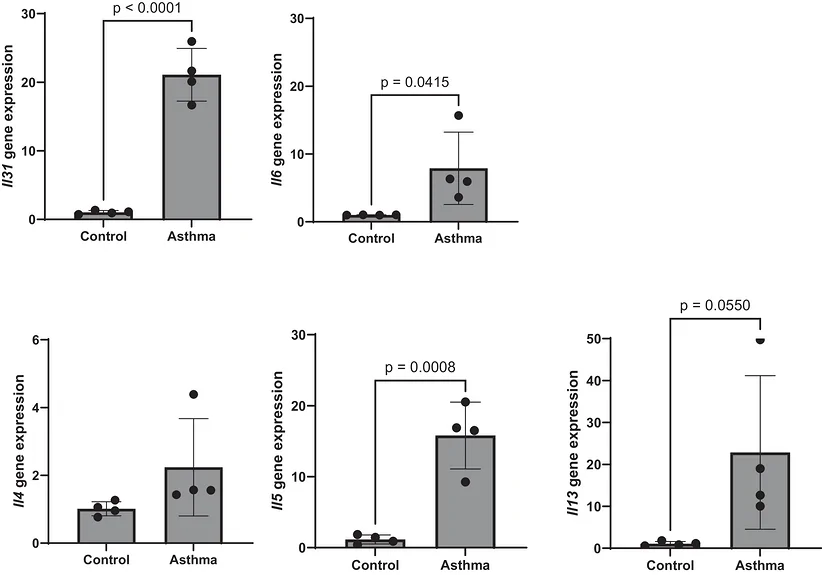
*Il‐31* expression levels are elevated in *Dermatophagoides farinae* (*Derf*)‐induced asthma model mice. Hilar lymph nodes were collected 24 h after the final *Derf* challenge. Gene expression levels of Th2 cytokines (*Il4*, *Il5*, and *Il13*)*, Il6*, and *Il3*1 were quantified via quantitative polymerase chain reaction (qPCR). Data represent mean ± SEM (*n* = 4). Statistical significance was assessed using an unpaired *t*‐test.

To evaluate whether IL‐31 participates in allergic airway inflammation in an allergic asthma mouse model induced by *Derf*, the most common HDM species, we quantified the Th2‐associated cytokine transcript levels in the hilar LNs of mice subjected to repetitive *Derf* exposure. Consistent with a robust Th2‐type response, *Il4*, *Il5*, *Il13*, and *Il6* mRNA levels were markedly elevated compared to those in control mice (Figure 2). Importantly, *Il31* levels were also significantly upregulated in the same tissues, suggesting activation of the IL‐31 axis in allergen‐induced asthma. Interestingly, *IL31* levels were also increased in HDM‐sensitized patients (Figure 1A). These results suggest that IL‐31 expression is induced in both murine and human allergic asthma and contributes to disease mechanisms beyond inflammation.

### 3.3 IL‐31 Deficiency Does Not Alter Airway Inflammation and Immune Cell Profiles

**FIGURE 3:**
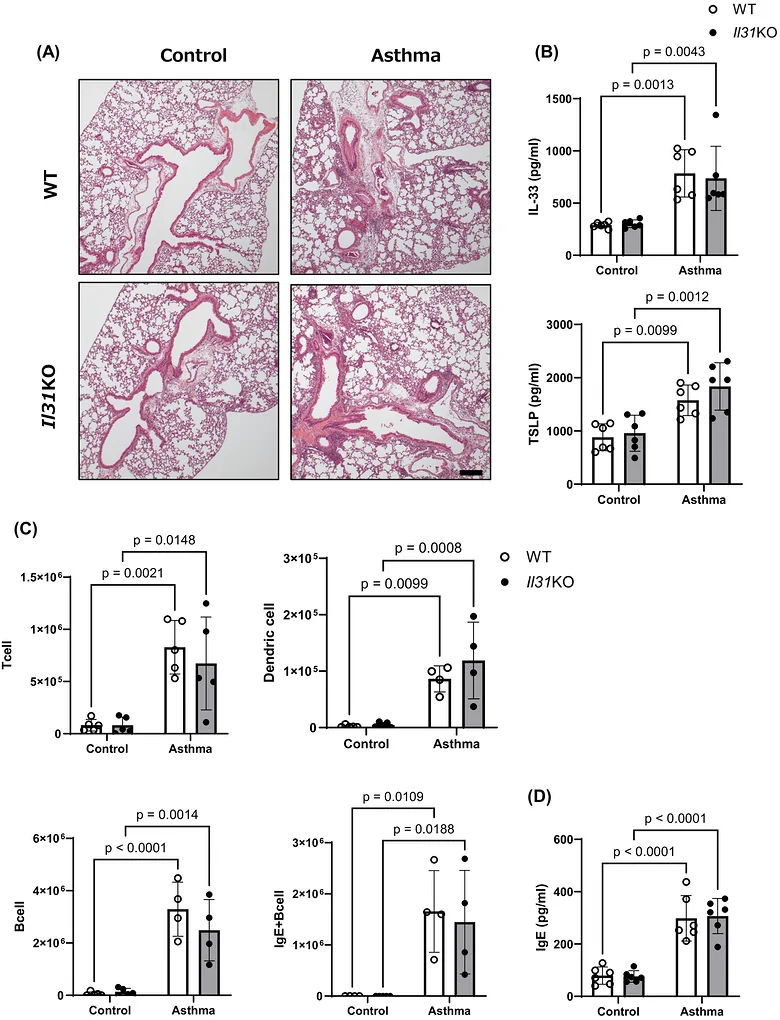
IL‐31 deficiency does not alter airway inflammation in *Derf*‐challenged mice. Wild‐type (WT) and Il31 knockout (KO) mice were subjected to *Derf*‐induced asthma. (A) Representative hematoxylin and eosin (H&E)‐stained lung sections showing comparable peribronchial and perivascular inflammatory infiltrates (scale bar = 100 µm). (B) Bronchoalveolar lavage fluid (BALF) concentrations of IL‐33 and thymic stromal lymphopoietin (TSLP) were measured via enzyme‐linked immunosorbent assay (ELISA). Data represent mean ± SEM (*n* = 6 per group). (C) Immune cell populations, including T, B, and dendritic cell populations, in hilar lymph nodes were analyzed via flow cytometry. Data represent mean ± SEM (T cells, *n* = 5 per group; B cells, *n* = 4 per group; dendritic cells, *n* = 4 per group). (D) Serum total IgE levels were measured via ELISA. Data represent mean ± SEM (*n* = 6 per group). Statistical significance was assessed via two‐way analysis of variance (ANOVA) with multiple comparisons.

To determine whether IL‐31 influences the immune and inflammatory responses in asthma, we compared WT and Il31 KO mice after *Derf* challenge. Histological examination of lung sections revealed comparable peribronchial and perivascular inflammatory infiltrates in both groups, indicating that IL‐31 deficiency did not attenuate airway inflammation (Figure 3A). Similarly, concentrations of epithelial‐derived cytokines IL‐33 and TSLP in BALF were not significantly different between WT and Il31 KO mice (Figure 3B). Moreover, the expression levels of Th2 cytokine genes (*Il4*, *Il5*, and *Il13*) in hilar LNs were not significantly different between WT and Il31 KO mice (Figure S1). Flow cytometric analysis of hilar LNs revealed similar distributions of CD4^+^ T cells, CD11b^+^ macrophages, CD11c^+^ dendritic cells, and CCR3^+^ eosinophils in both groups (Figure 3C). Serum total IgE levels were also comparable between WT and Il31 KO mice (Figure 3D). Collectively, these findings indicated that IL‐31 was dispensable for the development of Th2‐mediated airway inflammation under the tested conditions, prompting further investigation of the potential neural mechanisms underlying its function.

### 3.4 *Derf* Challenge Upregulates *Il31ra* and *F2rl1* (PAR‐2) Levels in VGs

**FIGURE 4:**
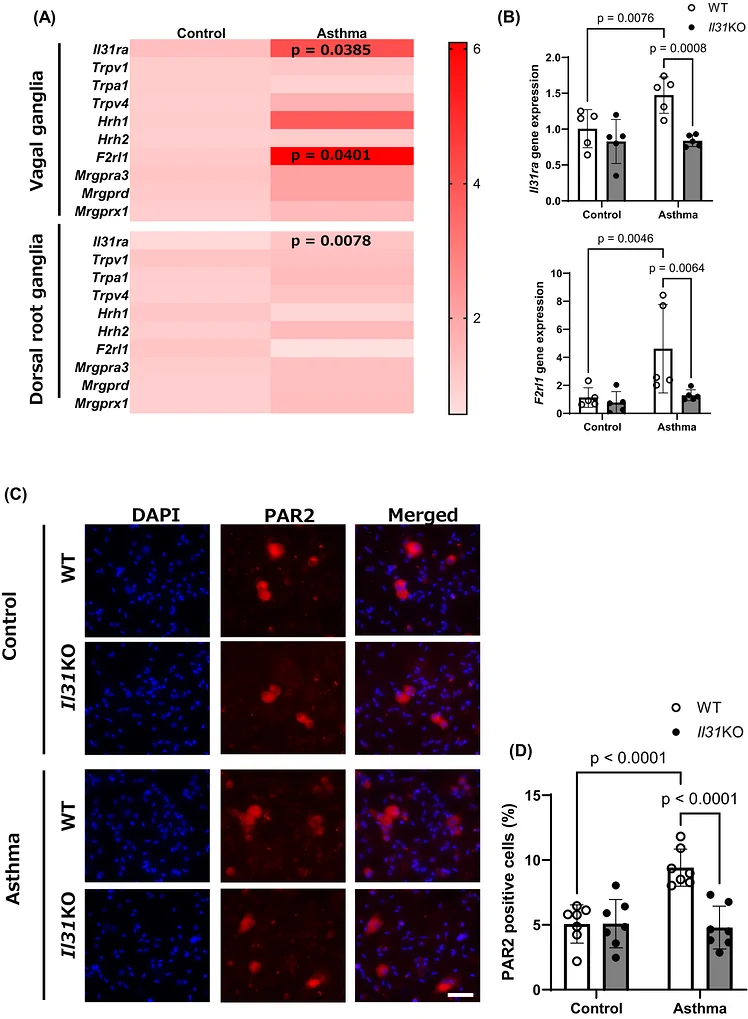
IL‐31 promotes neuronal protease‐activated receptor (PAR)‐2 expression in vagal ganglia. (A) qPCR analysis of *Il31ra* and *F2rl1* (PAR‐2) levels in the vagal (VG) and dorsal root (DRG) ganglia of *Derf*‐challenged mice. Data represent mean ± SEM (*n* = 3 per control group and *n* = 4 per asthma group). (B) Comparison of gene expression levels in VGs between WT and Il31 KO mice. Data represent mean ± SEM (*n* = 5 per group). (C) Representative immunofluorescence images of VG neurons stained for PAR‐2 (red), with 4',6‐diamidino‐2‐phenylindole (DAPI) as the nuclear counterstain (blue; scale bar = 50 µm). (D) Percentage of PAR‐2‐positive neurons in VGs. Data represent mean ± SEM (*n* = 7 per group). Statistical significance was assessed via two‐way ANOVA with multiple comparisons.

To explore the involvement of neuronal IL‐31 signaling, we examined *Il31ra* and *F2rl1* (PAR‐2) expression levels in the VGs and DRGs of *Derf*‐challenged mice. Both types of ganglia exhibited higher *Il31ra* expression levels than controls, with the upregulation being more pronounced in VGs, which house airway‐projecting sensory neurons (Figure 4A). Among cough‐related receptors, *F2rl1* levels were significantly increased in VGs, but not in DRGs, indicating the site‐selective induction of PAR‐2 in airway‐related sensory neurons.

Genotype comparison revealed significantly elevated *Il31ra* and *F2rl1* transcript levels in *Derf*‐challenged WT mice; however, this effect was not observed in *Il31* KO mice (Figure 4B). Immunofluorescence analysis confirmed these molecular findings: The proportions of PAR‐2‐positive neurons in VGs were markedly high in *Derf*‐challenged WT mice but remained unchanged in *Il31* KO mice (Figure 4C,D). These data suggest that IL‐31 signaling enhances PAR‐2 expression specifically in vagal sensory neurons during allergic airway inflammation.

### 3.5 IL‐31 Is Necessary for Allergen‐Induced Enhancement of PAR‐2 Responsiveness

**FIGURE 5:**
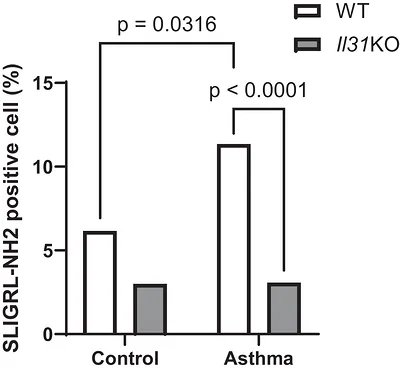
IL‐31 is necessary for allergen‐induced PAR‐2 neuronal responsiveness. VG neurons were loaded with Fura‐2 AM and stimulated with the PAR‐2 agonist SLIGRL‐NH_2_. Percentages of responsive neurons in WT and *Il31* KO mice under control and *Derf*‐challenged conditions are shown. Data were pooled from seven WT mice and eight *Il31* KO mice. Statistical significance was assessed using Fisher's exact test (two‐sided). Table 1 lists the total number of neurons analyzed and the number and percentage of neurons responding to SLIGRL‐NH_2_.

To determine whether IL‐31 influences the functional responsiveness of vagal neurons, we assessed Ca^2^^+^ influx following PAR‐2 activation using the agonist peptide SLIGRL‐NH_2_. In VG cultures from *Derf*‐challenged WT mice, the proportion of neurons responding to SLIGRL‐NH_2_ was significantly higher than that in untreated controls, indicating enhanced PAR‐2‐dependent excitability (Figure 5). In contrast, *Il31* KO mice did not exhibit this increase, with both the fraction of responding neurons and amplitude of Ca^2^^+^ transients being comparable to baseline levels (Figure 5 and Table 1). These findings suggest that IL‐31 is essential for the allergen‐induced sensitization of PAR‐2‐expressing vagal neurons.

**TABLE 1:** Ca^2^^+^ responses to PAR‐2 activation in vagal neurons from WT and *Il31* KO mice.

	CTL		Asthma	
	WT	*Il31*KO	WT	*Il31* KO
**Positive cells**	**18**	**10**	**36**	**10**
**Total cells**	**292**	**332**	**317**	**324**

## 4 Discussion

This study identified a previously unrecognized neuroimmune mechanism linking the Th2 cytokine IL‐31 to allergen‐induced cough in asthma. Using a *Derf*‐induced murine model, we found that IL‐31 levels were upregulated during allergic airway inflammation and acted not by modulating immune cell recruitment or cytokine production but by enhancing neuronal excitability in VGs via PAR‐2 induction. Our findings suggest that the IL‐31–PAR‐2–vagal axis contributes to cough hypersensitivity in allergic asthma, providing new insights into the neuroimmune regulation of airway sensory responses.

Previous clinical and experimental studies have reported increased IL‐31 levels in allergic disorders, such as atopic dermatitis, allergic rhinitis, and asthma (Akkenepally et al. 2023; Dillon et al. 2004; Huang et al. 2023). Consistently, we found that *Il31* transcript levels were upregulated in the hilar LNs of *Derf*‐challenged mice, paralleling the increased levels of Th2 cytokines, such as *Il4*, *Il5*, and *Il13*. Similarly, reanalysis of public gene expression datasets revealed higher IL‐31 expression levels in the CD4^+^ T cells of HDM‐sensitized patients with asthma than in those of healthy individuals. These findings suggest that IL‐31 production accompanies Type 2 immune activation in asthma and reflects a broader Th2 cytokine milieu rather than isolated signaling. Subsequent analyses revealed that IL‐31 played a functionally distinct role from canonical Th2 cytokines, acting primarily on sensory neurons rather than immune effector cells.

Despite the robust induction of *Il31* during *Derf*‐induced inflammation, *Il31* deficiency did not attenuate the classical hallmarks of allergic airway inflammation, including peribronchial infiltration, eosinophilia, and elevated serum IgE levels. Cytokines such as IL‐33 and TSLP, which drive Th2 polarization via epithelial activation, were also unaffected by IL‐31 loss. These results suggest that IL‐31 signaling is dispensable for the initiation and maintenance of airway inflammation under the tested conditions.

The above‐mentioned finding contrasts with previous reports that IL‐31 promotes inflammation via epithelial or macrophage activation in chronic dermatitis and fibrotic diseases (Akkenepally et al. 2023; Cevikbas et al. 2014; Ruzicka et al. 2017). This discrepancy possibly reflects tissue‐specific receptor expression patterns: IL‐31RA is highly expressed on cutaneous and neuronal cells but less abundant in airway epithelial and immune cells under homeostatic conditions. Our data suggest that IL‐31 does not directly influence Th2 immune pathways in the lungs but instead acts via neuronal mechanisms to modify the sensory reflexes associated with asthma symptoms, such as cough.

Notably, IL‐31 deficiency abolished allergen‐induced upregulation of *Il31ra* and *F2rl1* (PAR‐2) levels in VGs, which house airway‐projecting sensory neurons. Immunofluorescence analysis confirmed the increased proportion of PAR‐2‐positive neurons in *Derf*‐challenged WT mice, but this effect was not observed in *Il31* KO mice. Functionally, IL‐31 was necessary for enhanced neuronal responsiveness to the PAR‐2 agonist SLIGRL‐NH_2_, as evidenced by calcium imaging. Together, these results suggest that IL‐31 promotes both the transcriptional induction and functional sensitization of PAR‐2 in airway‐related sensory neurons.

PAR‐2 is implicated in cough and airway hypersensitivity through its activation by serine proteases derived from allergens, epithelial cells, and inflammatory cells (Amadesi et al. 2004; Dai et al. 2007). PAR‐2 activation leads to the depolarization of vagal afferent neurons via TRPV1 and TRPA1 channels, resulting in an exaggerated cough reflex (Kupari et al. 2019). By enhancing PAR‐2 expression and responsiveness, IL‐31 effectively lowers the threshold for neuronal activation in response to protease‐containing allergens, such as *Derf*. This mechanism provides a plausible explanation for how allergen exposure drives persistent cough, even when inflammation is controlled, as IL‐31‐dependent neuronal changes can sustain cough hypersensitivity independently of immune activity.

Our data revealed IL‐31 as a key cytokine bridging immune activation and sensory neural sensitization, a concept increasingly recognized in chronic allergic diseases. Traditionally, asthma pathogenesis has been viewed through the lens of airway inflammation and remodeling (Tiotiu et al. 2025). However, sensory neuronal involvement is now understood to contribute significantly to symptoms, including cough, dyspnea, and bronchoconstriction. IL‐31, through its receptor expression on neurons, possibly acts as a direct molecular link between Th2 cytokine networks and sensory hyperreactivity.

Similar neuroimmune interactions have been described in pruritic skin disorders, where IL‐31 directly activates IL‐31RA^+^ DRG neurons, leading to chronic itch (Cevikbas et al. 2014). The parallel between pruritus and cough is conceptually compelling: Both are protective reflexes mediated by peripheral sensory neurons, both become pathologically hypersensitive, and both are triggered by Th2 cytokine signaling. Our findings extend this paradigm to the airways, positioning IL‐31 as a functional analog of the “itch cytokine” in cough hypersensitivity.

PAR‐2 upregulation by IL‐31 possibly amplifies the effects of allergen‐derived proteases, creating a feed‐forward loop between Th2 cytokines and environmental triggers. This interaction potentially underlies the clinical observation that some patients with asthma experience disproportionate cough responses upon minimal allergen exposure, even when inflammation is mild. Targeting IL‐31 signaling possibly attenuates this hypersensitivity loop, offering therapeutic benefits beyond traditional anti‐inflammatory approaches.

Although IL‐31 is a classical Th2 cytokine, its functional outcomes differ markedly across tissues. In the skin, IL‐31 contributes to inflammation by inducing cytokine and chemokine production from keratinocytes and macrophages (Bilsborough et al. 2006; Kasraie et al. 2010). Our data suggest that its dominant role is neuromodulatory in the airways. This context‐dependent activity possibly reflects differential IL‐31RA expression and receptor coupling: IL‐31–signal transducer and activator of transcription 3 (STAT3) signaling pathway engages ion channels and excitatory pathways in neurons (Takahashi et al. 2023), but, in general, it activates the Janus kinase/STAT3‐, Janus kinase/STAT5‐, MEK/extracellular signal‐regulated kinase‐, and phosphoinositide 3‐kinase/Akt‐mediated transcriptional responses (Huang et al. 2022; Nemmer et al. 2021).

ERK1/2 and STAT3 signaling have been implicated in the regulation of *F2rl1*, which encodes PAR2. Indeed, injury‐induced upregulation of *F2rl1* mRNA has been shown to be attenuated by inhibition of either ERK1/2 or STAT3 (Benkafadar et al. 2024). As described above, binding of IL‐31 to its receptor complex activates several downstream signaling pathways, including the JAK–STAT and RAS–MEK–ERK pathways. Moreover, *Il31ra* and *F2rl1* have been reported to be co‐expressed in a subset of small‐diameter somatosensory neurons in mice (Hassler et al. 2020). Therefore, in the HDM‐induced asthma model used in the present study, IL‐31–IL‐31RA/OSMR signaling may induce or maintain F2rl1 expression in vagal sensory neurons through ERK‐ or STAT3‐dependent mechanisms. This hypothesis is consistent with our finding that HDM exposure increased PAR2 expression in WT mice, whereas this increase was absent in Il31 KO mice. Conversely, in a model of allergic skin inflammation, HDM exposure and *F2rl1* overexpression have been reported to increase Il31ra transcription in sensory neurons (Braz et al. 2021). Collectively, these findings suggest the possible existence of a positive feedback loop between the two pathways, in which PAR2‐associated signaling enhances IL‐31 responsiveness in sensory neurons, whereas IL‐31 receptor signaling promotes PAR2 expression. Such reciprocal amplification may produce a sustained increase in the responsiveness of vagal sensory neurons to protease‐mediated stimuli, including those derived from HDM, thereby contributing to airway sensory hypersensitivity and an exaggerated cough reflex. Nevertheless, further studies are required to determine whether IL‐31 directly induces *F2rl1* expression in vagal sensory neurons and to establish a causal relationship between this pathway and cough.

The absence of altered immune parameters in *Il31*‐deficient mice further underscored this divergence. Although IL‐31RA and oncostatin M receptor β are expressed on airway structural cells, their functional significance in asthma is limited compared to their well‐defined roles in pruritic disorders. The selective upregulation of *Il31ra* and *F2rl1* levels in VGs further supports a neuron‐centric mechanism. Therefore, IL‐31 is a unique cytokine that exerts organ‐specific effects by targeting sensory circuits rather than classical immune pathways.

Identification of the IL‐31–PAR‐2–vagal axis has important translational implications. Chronic cough remains a major unmet need in asthma management, and current treatments targeting inflammation or bronchoconstriction often fail to resolve cough hypersensitivity. IL‐31 or IL‐31RA blockade, which has been proven to be effective in atopic dermatitis through monoclonal antibodies such as nemolizumab, shows potential as an antitussive strategy for allergic asthma. By attenuating neuronal hyperexcitability, IL‐31RA inhibitors may reduce cough frequency and improve the quality of life, without impairing essential immune defenses.

Our findings suggest that IL‐31 and IL‐31RA levels in airway tissues can serve as biomarkers for cough‐predominant asthma phenotypes. Therefore, identifying patients with upregulated IL‐31 signaling may facilitate precision therapy targeting neuroimmune pathways rather than global inflammation.

This study has several limitations. First, although the *Derf*‐induced model recapitulated many features of human asthma, direct behavioral measurements of cough were not performed. Therefore, future studies using cough monitoring or electrophysiological recording are necessary to confirm the causal link between IL‐31 signaling and cough behavior. Second, the downstream molecular pathways by which IL‐31 enhances PAR‐2 expression remain unknown. Possible mechanisms include STAT3‐dependent transcriptional regulation or cross‐talk with the neuronal mitogen‐activated protein kinase and calcium signaling cascades. Third, whether IL‐31 acts directly on vagal neurons or indirectly via satellite glial or immune cells within ganglia remains unclear, warranting further investigation using cell‐specific receptor KO models.

Future studies should explore whether IL‐31 synergizes with other neuroactive cytokines, such as IL‐5, IL‐13, and TSLP, to orchestrate sensory neuron remodeling. Elucidating this broader neuroimmune network may reveal new combinatorial therapeutic targets for cough and airway hyperreactivity in allergic diseases.

## 5 Conclusions

In summary, this study found that IL‐31, a Th2 cytokine best known for its role in pruritus, mediated allergen‐driven cough via a distinct neuronal mechanism. Specifically, IL‐31 upregulated its receptor and PAR‐2 in vagal sensory neurons, enhancing their responsiveness to allergen proteases and sensitizing the cough reflex. Unlike other Th2 cytokines, IL‐31 did not significantly modulate airway inflammation but instead acted on the neuronal components of allergic responses. These findings highlight the IL‐31–PAR‐2–vagal pathway as a novel neuroimmune circuit underlying cough hypersensitivity in asthma and suggest IL‐31 signaling as a promising therapeutic target for cough‐predominant allergic airway disease.

## Author Contributions

**Takayoshi Miyamoto**: conceptualization, methodology, data curation, investigation, validation, formal analysis, visualization, project administration, resources, writing – review and editing. **Chiharu Ohira**: methodology, investigation, writing – review and editing. **Mao Kaneki**: methodology, investigation, writing – review and editing. **Mariko Komuro**: investigation, methodology, writing – review and editing. **Mana Ichikawa**: methodology, investigation, writing – review and editing. **Ibuki Yasuda**: methodology, investigation, writing – review and editing. **Yoshiichi Takagi**: methodology, investigation, writing – review and editing. **Tomoki Fukuyama**: conceptualization, methodology, software, data curation, investigation, validation, formal analysis, supervision, visualization, project administration, resources, writing – original draft, writing – review and editing.

## Funding

The authors have nothing to report.

## Conflicts of Interest

Yoshiichi Takagi is an employee of Japan SLC, Inc. The other authors declare no conflicts of interest.

## Supporting information




**Figure S1**: IL‐31 deficiency does not alter Th2 cytokine gene expression in Derf‐challenged mice. Hilar lymph nodes were collected 24 h after the final Derf challenge. Gene expression levels of Th2 cytokines (*Il4*, *Il5*, and *Il13*) were quantified by quantitative polymerase chain reaction (qPCR). Data represent mean ± SEM (n = 4 per group).

**Table S1**: List of Primers Utilized in This Study

## Data Availability

The data supporting the findings of this study are available from the corresponding author upon reasonable request.
