# Supplementary material for: Roles of Interleukin‐31 in Allergen‐Driven Cough: Linking Protease‐Activated Receptor‐2 Activation and Vagal Ganglion Sensitization in a Dermatophagoides farinae‐Induced Asthma Model
Source: Brain Behav. 2026 Sep 4;16(9):e71731. doi: 10.1002/brb3.71731 (PMC13544625; doi:10.1002/brb3.71731)
Supplement: Supplementary file 1 [file BRB3-16-e71731-s001.pdf]

Fig. S1

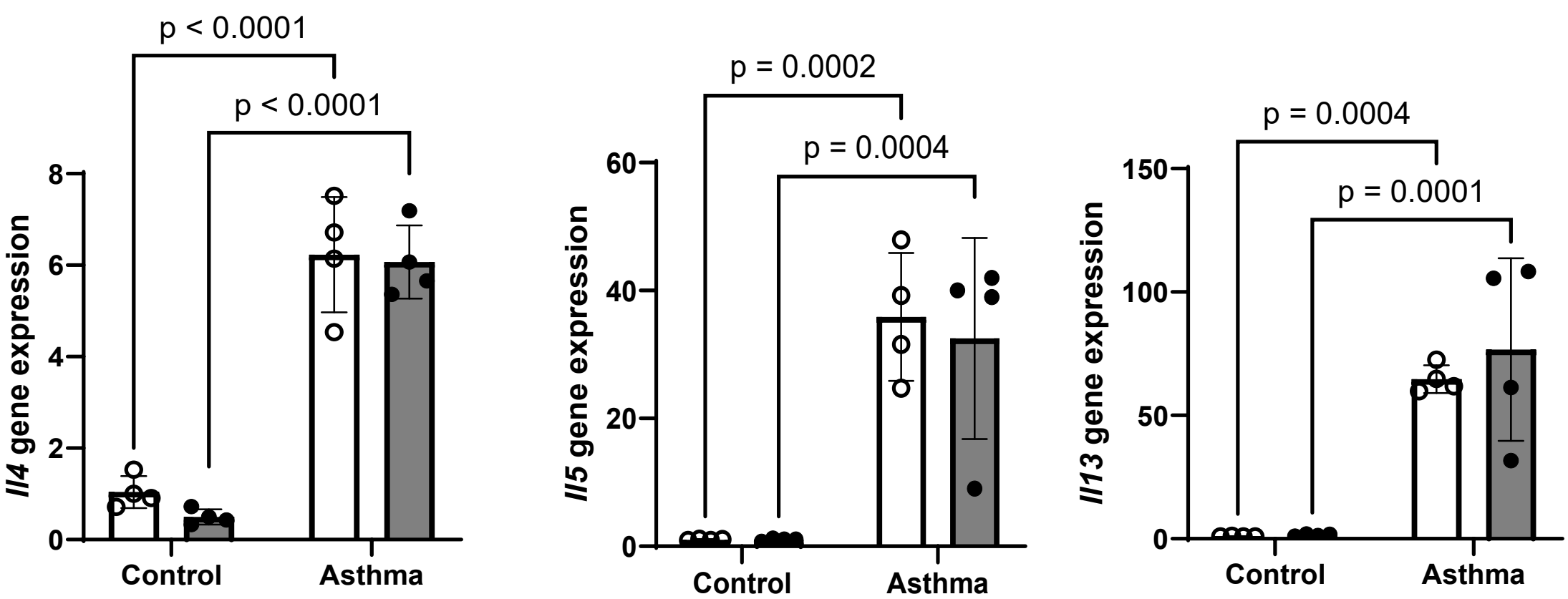

p < 0.0001
p < 0.0001
Il4 gene expression
0
2
4
6
8
Control
Asthma
p = 0.0002
p = 0.0004
Il5 gene expression
0
20
40
60
Control
Asthma
p = 0.0004
p = 0.0001
Il13 gene expression
0
50
100
150
Control
Asthma
