# Supplementary material for: Roles of Interleukin‐31 in Allergen‐Driven Cough: Linking Protease‐Activated Receptor‐2 Activation and Vagal Ganglion Sensitization in a Dermatophagoides farinae‐Induced Asthma Model
Source: Brain Behav. 2026 Sep 4;16(9):e71731. doi: 10.1002/brb3.71731 (PMC13544625; doi:10.1002/brb3.71731)
Supplement: Supplementary file 2 [file BRB3-16-e71731-s002.docx]

Table S1. List of Primers Utilized in This Study
